# Exploring high-order correlations with deep-broad learning for autism spectrum disorder diagnosis

**DOI:** 10.3389/fnins.2022.1046268

**Published:** 2022-11-22

**Authors:** Xiaoke Hao, Qijin An, Jiayang Li, Hongjie Min, Yingchun Guo, Ming Yu, Jing Qin

**Affiliations:** ^1^School of Artificial Intelligence, Hebei University of Technology, Tianjin, China; ^2^School of Nursing, Centre for Smart Health, The Hong Kong Polytechnic University, Hong Kong, Hong Kong SAR, China

**Keywords:** autism spectrum disorder, high-order functional brain network, broad learning system, classification, feature selection

## Abstract

Recently, a lot of research has been conducted on diagnosing neurological disorders, such as autism spectrum disorder (ASD). Functional magnetic resonance imaging (fMRI) is the commonly used technique to assist in the diagnosis of ASD. In the past years, some conventional methods have been proposed to extract the low-order functional connectivity network features for ASD diagnosis, which ignore the complexity and global features of the brain network. Most deep learning-based methods generally have a large number of parameters that need to be adjusted during the learning process. To overcome the limitations mentioned above, we propose a novel deep-broad learning method for learning the higher-order brain functional connectivity network features to assist in ASD diagnosis. Specifically, we first construct the high-order functional connectivity network that describes global correlations of the brain regions based on hypergraph, and then we use the deep-broad learning method to extract the high-dimensional feature representations for brain networks sequentially. The evaluation of the proposed method is conducted on Autism Brain Imaging Data Exchange (ABIDE) dataset. The results show that our proposed method can achieve 71.8% accuracy on the multi-center dataset and 70.6% average accuracy on 17 single-center datasets, which are the best results compared with the state-of-the-art methods. Experimental results demonstrate that our method can describe the global features of the brain regions and get rich discriminative information for the classification task.

## Introduction

Autism spectrum disorder (ASD) is a neurologically heterogeneous disorder that is difficult to diagnose. The main characteristics of ASD patients are social interaction disorders and neurodevelopmental disorders of stereotyped behavior. The life expectancy of ASD patients is much lower than that of normal controls (NC) (Perkins and Berkman, 2012). The current psychiatric diagnosis for ASD refers only to symptomatic behavioral observations (DSM-5/ICD-10), which may be misdiagnosed (Nickel and Huang-Storms, 2017). However, the cause and pathogenesis of ASD are unclear. There is an urgent need to identify biomarkers associated with brain imaging data to assist medical diagnosis.

Recently, various non-invasive brain imaging techniques such as magnetic resonance imaging (MRI), positron emission tomography (PET), and functional magnetic resonance imaging (fMRI) are widely used in the study of neurodegenerative diseases such as ASD. In particular, several studies in recent years have shown that using the blood oxygen level-dependent (BOLD) signal as a neurophysiological indicator can effectively identify potential biomarkers in ASD patients (Dekhil et al., 2018). Many studies have been conducted based on low-order brain functional connectivity obtained from fMRI, which reflects the correlation relationship between signals from paired brain regions (Liang et al., 2012; Li et al., 2014). Due to the spontaneous aberrations generated in the functional connectivity status of brain disease patients (Hahamy et al., 2015), there is a significant variability compared to NC.

Many studies explore the low-order brain functional connectivity to diagnose ASD. Stacked multiple sparse autoencoders (SSAE) is applied to learn the discriminative feature representation of low-order brain functional connectivity and subsequently diagnose ASD (Kong et al., 2019). Dekhil et al. (2018) construct an ASD diagnostic system consisting of sparse autoencoders and spatially activated regions, which similarly learn low-order brain functional connectivity features. Wang et al. (2020) propose a multi-site domain adaptation method based on low-order brain network for ASD diagnosis. Wang et al. (2022) propose a multi-site clustering and nested feature extraction method for fMRI-based ASD detection. However, current methods only reflect correlations between pairs of brain regions. The connections between brain regions are complex, and studies that only reflect pairwise relationships between brain regions are still limited. In contrast to the traditional approaches to characterize lower-order brain functional connectivity (Yu et al., 2017; Li et al., 2019), high-order feature representation of brain connectivity can characterize complex patterns of interactions between multiple brain regions and correlations across brain regions. Feng et al. (2020) regard the second-order functional connectivity network as a higher-order brain network, in which brain connectivity patterns are only obtained by repeatedly computing first-order correlations between pairs of brain regions, and some features may be lost in the process of repeated computing. Gao et al. (2020) exploit clustering of functional connectivity time series to reveal high-order relationships among multiple regions of interest (ROIs), but global brain functional connectivity features have not been considered.

To overcome the above-mentioned drawbacks and to form high-order feature information that can characterize the global structure of the brain, we introduce the hypergraph structure to inscribe the high-order brain functional connectivity. Hypergraph (Ktena et al., 2018) is a novel tool for inscribing high-order structures, and the features of the hypergraph structure are distinguished from the traditional graph structure features. Unlike normal graphs, hypergraphs are composed of nodes and hyperedges. One hyperedge can connect two or more nodes. Hypergraph learning is flexible and powerful in modeling complex data dependencies such as brain networks. It has received more attention that using hypergraph to describe the brain connection pattern can more accurately describe the complex high-order connection relationship of the brain network.

Due to the complex features of brain networks, several recent studies use deep learning-based approaches to diagnose patients with autism spectrum disorders (Ktena et al., 2018; Gao et al., 2020; Yao et al., 2021). For example, Eslami et al. (2019) propose a self-encoder-based model for ASD classification. Heinsfeld et al. (2017) use two stacked denoising autoencoders to identify ASD patients from fMRI data. Xing et al. (2019) propose a novel convolutional neural network with elemental filters for the diagnosis of ASD. Huang et al. (2021) use Long Short-Term Memory Networks (LSTM) for the classification of ASD patients. Guo et al. (2017) and Khodatars et al. (2021) propose a deep neural network model for the study and diagnosis of ASD patients. Zhang et al. (2022) propose a feature selection method based on variational autoencoder pre-training using a multilayer perceptron for ASD classification. Jiang et al. (2020) propose a hierarchical GCN framework to learn brain network graph feature embeddings while considering both network topology information and subject associations. However, all these deep learning-based methods and graph neural network-based methods are based on low-order brain functional connectivity networks for subsequent feature extraction and classification, which have non-negligible drawbacks. Firstly, there are limitations in using low-order brain network features to represent brain connectivity patterns. Secondly, the models based on deep learning will become more complex as the number of model layers increases, the training process is time-consuming and the deep network features are not scalable. The number of parameters to be learned is huge, and it often faces the problem of insufficient single-center data, resulting in overfitting. It is not until the emergence of the Broad Learning System (BLS) (Chen and Liu, 2018) that traditional artificial intelligence methods are revolutionized. It represents a step towards building more effective machine learning methods that can further extend models based on deep learning methods and improve the learning efficiency of the models (Chen and Liu, 2018; Gong et al., 2022). Recently, some studies have introduced BLS and its variant algorithms into medical image analysis (Han et al., 2020), providing an effective tool for diagnosing AD in MRI images. However, there are no studies based on BLS to diagnose ASD in fMRI. Benefiting from the superiority of BLS, we use it for further feature selection and classification.

Compared with traditional deep learning-based diagnostic models, our proposed deep-broad learning method can learn complex and high-dimensional brain connectivity network features more accurately. We use the functional connectivity and hypergraph structure of fMRI to characterize the high-order connectivity characteristics of the brain. The feature learning process is further extended by using the structure fused by the autoencoder and the BLS, and an efficient and accurate brain network learning structure is obtained. The main contributions of this paper are summarized as follows:

Firstly, we construct a high-order brain functional connectivity network of the functional connectivity structure of fMRI based on hypergraph structure, which improves the ability of traditional brain functional connectivity networks to express brain structure.

Secondly, we propose a novel combinatorial deep-broad learning method to extract high-dimensional discriminative features of high-order brain functional connectivity networks.

Compared with other ASD classification models, our model not only takes into account the global functional connectivity features of the brain but also provides a feature-learning classification module with fewer parameters using BLS.

The purpose of this paper is to propose an effective model for portraying the global functional connectivity structure of the brain. The BLS further enhances the feature learning capability and computational speed of deep learning models for ASD diagnosis. The rest of the paper is structured as follows. In section 3, we introduce the dataset materials and the details of our proposed method. In section 4, we perform an experimental evaluation and experimental analysis of the proposed method. In section 5, we summarize the work presented in this paper.

## Materials

In this study, we use 505 patients with ASD and 530 healthy controls from 17 sites in the ABIDE- I [ABIDE (http://fcon_1000.projects.nitrc.org/indi/abide/)] dataset for our experiments. Our study uses data pre-processed by the C-PAC pipeline (Agastinose Ronicko et al., 2020) with the following pre-processing processes: motion correction, slice timing correction, removal of interfering signals, low-frequency drift and voxel intensity normalization. ABIDE provides a variety of ROI segmentation options. In this study, we use 200 uniform ROIs generated by the spatially constrained spectral clustering algorithm (Craddock et al., 2012).

## Method

**FIGURE 1:**
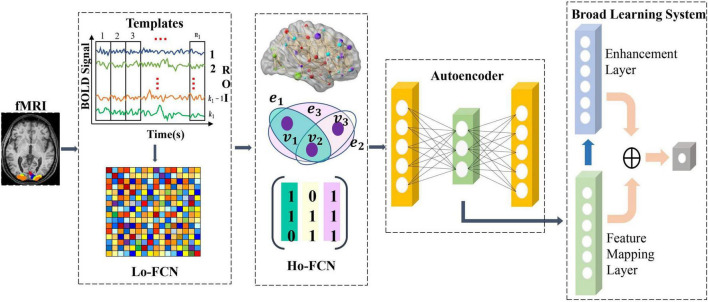
The illustration of our proposed method.

We propose a deep-broad learning method to explore high-order brain functional connectivity network features for ASD classification. The specific structure of the model is shown in Figure 1. The model consists of four parts. (1) Firstly, the low-order brain functional connectivity network is constructed by calculating the time-series Pearson correlation matrix of the fMRI data. It is used to portray the low-order local features of the brain shown in Figure 1 as Lo-FCN. (2) We introduce the hypergraph structure to construct high-order brain functional connectivity network to inscribe the global features of the brain shown in Figure 1 as Ho-FCN. (3) Initial feature learning of high-dimensional high-order brain functional connectivity network is performed using an autoencoder. (4) Finally, the initial features learned by autoencoder are fed into the BLS for further learning and classification. The details of each step will be given in the following sections.

### Construction of high-order brain functional connectivity network

We obtain the high-order brain functional connectivity network based on time series of fMRI and coactivation level signals based on hypergraph to effectively characterize the global brain connectivity pattern. Specifically, we first calculate the correlations between pairs of brain regions using Pearson correlation coefficients, which are widely used to calculate the functional connectivity of fMRI (Liang et al., 2012; Baggio et al., 2014; Zhang et al., 2017), as shown in Equation (1). *u* and *v* represent the time series of two ROIs, the length of each series is *T*, $\bar{u}$ and $\bar{v}$ represent the average values of the time series *u* and *v*, respectively. Calculating the pairwise correlation of all time series will get the paired brain regions correlation matrix *Corr*_*M*×*M*_, where *M* is the number of ROIs, so as to obtain the low-order brain functional connectivity network.


(1) $$\rho_{u\,v}=\frac{\sum_{t=1}^{T}\left({\text{u}}_{t}-\bar{\text{u}}\right)\,\left({\text{v}}_{t}-\bar{\text{v}}\right)}{\sqrt{\sum_{t=1}^{T}{\left({\text{u}}_{t}-\bar{\text{u}}\right)}^{2}}\,\sqrt{\sum_{t=1}^{T}{\left({\text{v}}_{t}-\bar{\text{v}}\right)}^{2}}}$$

Each element in the low-order brain functional connectivity matrix depicts the correlation between local pairwise ROIs. When the paired ROIs are highly correlated, the element value approaches 1, and when the paired brain interval is inversely correlated, the element value is close to -1. Since pairwise relationships between brain regions only characterize local features of the brain, we use the hypergraph to represent correlations in the interaction of multiple brain regions rather than pairwise correlations, resulting in a hypergraph-based high-order brain network. Specifically, we first recall the basics of hypergraph (Schölkopf et al., 2007), where we denote a hypergraph as *G* = {*V*,*E*,*W*}, the set of hypergraph vertex of the hypergraph *V* = {*v*_1,_*v*_2_,…,*v*_*n*_} represents the *n* brain regions, hyperedge *E* = {*e*_1,_*e*_2_,…,*e*_*m*_} represents the correlation between brain regions, each hyperedge is assigned a weight *w*(*e*_*i*_),1 ≤ *i* ≤ *M*. The weight vector of a hyperedge is expressed as *W* = {*w_e_*_1_,*w_e_*_1_,…,*w_e_*_*m*_}. The hypergraph structure can be represented simply as an association matrix *H* ∈ {0,1}^|*V*| × |*E*|^, each element in *H* indicates whether the vertex *v* is in the hyperedge *e*,denoted as $H\left(v,e\right)=\;\{\begin{array}{c} 1\;if\;v\in e\\ \;0\;if\;v\notin e\; \end{array}$. The elements in the association matrix represent the probability value of the importance of the node to the hyperedge. Based on the constructed association matrix, the degree of the hypergraph node and the degree of the hyperedge can be obtained, which are expressed as: *d*(*v*_*i*_) = ∑_*ej∈ε*_ *w_ej_H*_*ij*_ for 1 ≤ *i* ≤ *N*, δ (*e*_*j*_) = ∑_*vi∈v*_*H*_*ij*_ for 1 ≤ *j* ≤ *M*. The degree matrix of the hypergraph vertex and the hyperedge are described as *Degree*_*e*_ ∈ *R*^|*E*| × |*E*|^ and *Degree*_*v*_ ∈ *R*^|*V*| × |*V*|^. *D*_*e*_ ∈ *R*^|*E*| × |*E*|^and *D*_*v*_ ∈ *R*^|*V*| × |*V*|^ are the diagonal matrices containing the hypergraph vertex and the hyperedge. In graph theory, the graph Laplacian matrix plays an important role in graph learning. Based on the graph Laplacian matrix, by calculating its eigenvalues and eigenvectors, According to previous research (Schölkopf et al., 2007), we can perform a spectral analysis of the graph. For simple graphs, the graph Laplacian is defined as △ = *D* − *A*, *D* is the diagonal matrix of vertex degrees, and *A* is the adjacency matrix, while for hypergraphs, the graph Laplacian is defined as △=Dv-H⁢W⁢De-1⁢HT. The normalized Laplace matrix is △=I-Dv-12⁢H⁢W⁢De-1⁢HT⁢Dv-12. We summarize the symbols and definitions in Table 1.

**TABLE 1:** Symbols and definitions of hypergraph.

Notation	Definition
*G* = {*V*,*E*,*W*}	*G* represents the hypergraph, *V*,*E*,*W* represent the set of vertices, the set of hyperedges, and diagonal matrix of hyperedge weights. respectively.
*d*(*v*_*i*_)	The degree of vertex *v*_*i*_.
*w*(*e*_*i*_)	The weight of hyperedge *e*_*i*_.
δ(*e*_*j*_)	The degree of hyperedge *e*_*j*_.
*H*	The |*V*| × |*E*| incidence matrix of hypergraph structure. *H*(*v*,*e*) indicate the connection strength between the vertex v and the hyperedge *e*.
*D*_*v*_	The diagonal matrix of vertex degrees.
*D*_*e*_	The diagonal matrix of hyperedge degrees.
△	The Laplacian matrix of hypergraph.

In order to obtain a hypergraph-based high-order brain functional connectivity network, we construct the hypergraph using the method proposed by the previous work (Schölkopf et al., 2007). We treat each hypergraph node as a brain region and use each brain region as a central region to calculate the Euclidean distance between the selected central region and other brain regions. Specifically, we first take each vertex (ROI) as a center node and calculate the Euclidean distance between the center and other vertices. Then we construct a hypergraph by connecting the center and its *K* nearest vertex. We regard the *k* brain regions closest to the central node Euclidean space $d_{i\,j}={\left|v_{i}-v_{j}\right|}_{2}^{2}$ as the nearest neighbors of central brain region. We refer to correlations between nodes as a hyperedge, *d*_*ij*_ represents the Euclidean distance between brain region *v_i* and *v_j*. Based on the above mentioned process, we construct the hypergraph based high-order brain connectivity network to represent the global features of brain.

### The novel feature extraction and classification method based on deep-broad learning

Due to the high dimensionality of the constructed high-order brain functional connectivity network features, we utilize a non-linear dimensionality reduction approach to reduce the feature dimension. Specifically, we use an autoencoder to reconstruct the features of the constructed hypergraph-based high-order brain feature representation. We obtain low-dimensional discriminative features of the high-order brain network by minimizing the reconstruction error between the input network features and the output features through self-supervised learning. The autoencoder consists of an encoder and a decoder.


(2) =e⁢n⁢c ∅e⁢n⁢c (x)=τ (We⁢n⁢cx+be⁢n⁢c)

We use the original high-order brain feature representation *x* as input to the autoencoder to obtain discriminative lower-dimensional feature *h*_*enc*_ via the encoder, denoted as Equation (2) where τ is the hyperbolic tangent activation function (*tanh*), and *W*_*enc*_ and *b*_*enc*_ represent the weight matrix and bias of encoder. Once we have obtained the low-dimensional feature representation *h*_*enc*_ of the high-order brain function connectivity network, we use the decoder to reconstruct the original input data *x*, expressed as Equation (3). The low-dimensional feature representation *h*_*enc*_ is input into the decoder, where *W*_*dec*_ and *b*_*dec*_ represent the weight matrix and bias of the decoder, respectively. We use Mean Squared Error (MSE) as the reconstruction loss, which represents the discrepancy between the reconstructed brain function connectivity network features *x*′ and the original features *x*. After completing the training of the autoencoder, we obtain the low-dimensional feature representation of the new high-order brain functional connectivity network as the effective feature. And we use it as the valid discriminative high-order brain function connectivity feature input broad learning system for further learning.


(3) $$\begin{array}{c} x^{\prime }=\emptyset_{d\,e\,c}\,\left(h_{e\,n\,c}\right)=W_{d\,e\,c}\,h_{e\,n\,c}+b_{d\,e\,c} \end{array}$$

**FIGURE 2:**
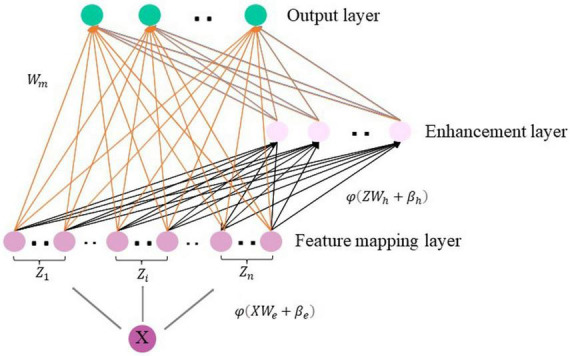
The structure of the broad learning system.


Analyzing higher-order brain functional connectivity using existing machine learning methods is challenging due to the high-dimensional, large-scale, and complex interdependencies between brain regions. Moreover, a large number of iterative processes during traditional model training requires huge amounts of time and computational resources. The Broad Learning System (BLS) (Chen and Liu, 2017, 2018; Chen et al., 2019) has recently become one of the most popular networks due to its excellent performance in machine learning tasks (Gong et al., 2022). BLS can map samples to a more suitable space to handle the large volume of high order brain functional network features and is suitable for processing time-varying data. BLS first map the inputs to construct a set of mapped features. A group of mapping nodes defined in our work is a mapped feature in original BLS. Given that the feature extraction at this step uses randomly generated weights, calculating multiple mapping features can enhance the stability of the extracted feature information and simplify the operations. Figure 2 illustrates the basic structure of the BLS, which consists of a three-layer network defined as the feature mapping layer, the enhancement layer and the output layer, where *X* ∈ *R^N^*^×*m*^ denotes the discriminative high-order brain function connectivity matrix learned by autoencoder, which is taken as the input to the BLS. *N* is the number of samples, and *m* is the feature dimension of each sample, *Y* ∈ *R^N^*^×*c*^(*c* < *m*) is the output layer of BLS, *c* is the feature dimension after the feature extraction by BLS of each sample, and *W*_*BLS*_ is the weight of the feature mapping layer and the enhancement layer to the output layer. Specifically, we first input the high-order brain function connectivity matrix *X* to the feature mapping layer to generate the *i*-th group of mapping nodes *Z_i*, denoted as Equation (4):

(4) $$\begin{array}{c} Z_{i}=\emptyset \,\left(X\,W_{e\,i}+\beta_{e\,i}\right),i=1,\ldots ,n \end{array}$$

*W*_*ei*_ and β_*ei*_ are the weight and bias from *X* to the feature mapping layer. Similarly, the *m*-*th* group of enhancement layer nodes *H_m* is generated by taking the mapping node as the input of the enhancement layer, which is expressed as Equation (5):


(5) $$\begin{array}{c} H_{m}\,\delta \equiv \left(Z^{n}\,W_{h_{m}}+\beta_{h_{m}}\right) \end{array}$$

*W*_*hm*_ and *β_hm_* are the weights and biases from the feature mapping layer to the enhancement layer. It should be noted that ∅ and δ are nonlinear functions, such as *tanh* and *tansig*, we compose all mapping nodes as *Z* = [*Z*_1_, *Z*_2_, …, *Z*_*n*_], and enhance the nodes as *H* = [*H*_1_, *H*_2_, …, *H*_*m*_].

The BLS model can be expressed as Equation (6):


$$Y=\left[Z_{1},\;Z_{2},\ldots ,Z_{n}|\delta \;\left(Z^{n}\,W_{h_{1}}+\beta_{h_{1}}\right),\ldots ,\;\delta \;\left(Z^{n}\,W_{h_{m}}+\beta_{h_{m}}\right)\right]$$

$$W_{B\,L\,S}=\left[Z_{1},Z_{2},\ldots ,Z_{n}|H_{1},H_{2},\ldots ,H_{m}\right]\;W_{B\,L\,S}$$

(6) $$\begin{array}{c} =\left[Z|H\right]\;W_{B\,L\,S} \end{array}$$

To summarize, we further learn the features extracted by autoencoder via BLS and finally get the ASD classification result.

### Experiments

In this section, we conduct two-stage experiments of our proposed method. In the first stage, we conduct experiments on 1,035 samples from 17 multi-centers and each single-center to demonstrate the effectiveness of our proposed method. In the second stage, we compare with the state-of-the-art methods using another atlas that divides the brain into 264 ROIs. The robustness and scalability of our proposed method are further verified by experiments on multi-atlas data.

In our experiments, we use 10-fold cross-validation to evaluate the classification accuracy of the prediction model. This means that we first randomly divide the dataset into 10 disjoint subsets of data, and then select a single subset as the test set, with the remaining 9 subsets used as the training set. In particular, for multi-center experiments, we mixed all samples from 17 centers, and then divided the dataset into 10 disjoint subsets. We selected 1 of the *K_e* parts as the test set and the remaining *K_e*-1 parts as the training set, finally took the average of the *K_e* verification results as the verification error of this model. The process is repeated 10 times to reduce the effect of sampling bias on the experimental results. The classification performance of the model is evaluated by comparing the accuracy (ACC), sensitivity (SEN), and specificity (SPE), and the mean of the experimental results for the single-center data is also calculated. Accuracy measures the proportion of subjects correctly classified (*i.e.*, actual ASD is classified as ASD and actual healthy is classified as healthy). Sensitivity represents the proportion of actual ASD subjects correctly classified as ASD, and specificity measures the proportion of actual healthy subjects classified as healthy. The running time means the training time and the inference time.

#### Experiments settings

The classification accuracy of our proposed model may be affected by a variety of parameters, including: (1) the choice of hypergraph parameters when constructing high-order brain functional connectivity networks, (2) the number of layers of autoencoders in the initial feature selection process, (3) the number of BLS nodes and the window size of each layer. The hypergraph parameters of the model include the nearest neighbor size *K* obtained based on the hypergraph similarity matrix. The number of autoencoder layers *L*, the number of nodes in the enhancement layer *E*, the mapping layer *M* and the window size *W* of the BLS are adjusted during the experiment. In our experiments, we adjust all free parameters by 10-fold cross-validation on the training set. Taking into account the effect of the hypergraph construction parameters, we optimize *K* in the range {4,5,…,12}. Since there is difference in the high-order brain features that can be learned by different layers of the autoencoder, we test the effect of different layers of the autoencoder on the experimental results for single-center and multi-center data, respectively. We adjust the number of layers of the autoencoder in the range {1,2,…,6}. In addition, the number of nodes in the mapping layer and enhancement layer of the BLS and the size of the window also have a significant impact on the classification results, so we test the classification performance under different node settings. We finally find that the hypergraph-based network of high-order brain function connections are constructed with *K* set to be 5. The number of layers of the autoencoder *L* is set to be 3. For the multi-center data, we set the parameters as *M* = 20, *E* = 10, *W* = 100. For the single-center data, we set the parameters as *M* = 200, *E* = 50. Depending on the optimal parameters chosen, we can obtain the best experimental results.

#### Classification performance

We compare our proposed method with: (1) support vector machine (SVM) with RBF kernel, (2) random forests, (3) deep neural network (DNN), (4) Autoencoder, four state-of-the-art methods shown in Table 2.

**TABLE 2:** Classification performance of different methods on multi-centers dataset.

Method	Accuracy (%)	Sensitivity (%)	Specificity (%)	Precision (%)	Running time(s)
SVM	60.3 ± 3.6	35.3 ± 8.2	84.4 ± 6.6	64.3 ± 5.9	186
Random forest	63.7 ± 7.2	54.9 ± 3.9	71.3 ± 9.2	68.9 ± 3.8	67
DNN	60.7 ± 5.4	56.4 ± 7.3	64.8 ± 3.8	70.1 ± 5.6	108030
Autoencoder	65.4 ± 6.6	69.3 ± 4.2	61.9 ± 5.2	60.8 ± 3.7	21600
Ours	71.8 ± 4.2	70.8 ± 3.8	65.9 ± 4.2	65.9 ± 4.8	1200

In the first part of the experiment, the results of the multi-center data are shown in Table 2. Experimental results demonstrate that our proposed method considers the efficiency of the model while maintaining accuracy. Moreover, the time required for ten-fold cross-validation was significantly reduced. Based on the experimental results, it is demonstrated that the high-order brain functional connectivity network constructed based on hypergraphs can capture more correlations between brain regions than the traditional lower-order brain functional connectivity network, and thus obtain more discriminative features. We confirm that BLS further learns the features extracted by autoencoder and greatly reduces the training time. Our method achieves 71.8% accuracy on multi-center data. Experiments show that our proposed method outperforms other state-of-the-art algorithms in terms of accuracy and training time.

At the same time, in order to verify the effectiveness of the proposed method on independent single-center data, we further conduct experiments on 17 single-center datasets. Table 3 shows the results of our experiments, which demonstrate that our method achieves better classification results on small sample datasets compared to the existing state-of-the-art methods. In particular, for the USM dataset, the accuracy is as high as 81.6%. The experiments demonstrate the significant superiority of our proposed method on small sample data as well.

**TABLE 3:** Classification performance of our proposed method on single-center dataset.

Sites	Accuracy (%)	Sensitivity (%)	Specificity (%)	Precision (%)
NYU	71.4 ± 5.7	75.0 ± 1.5	68.4 ± 9.4	66.7 ± 9.8
OHSU	80.8 ± 3.8	76.9 ± 7.7	84.6 ± 7.7	83.3 ± 8.3
KKI	62.5 ± 8.3	63.6 ± 9.1	61.5 ± 7.7	58.3 ± 8.4
YALE	71.4 ± 3.6	74.0 ± 2.9	68.9 ± 4.4	68.9 ± 2.5
USM	81.6 ± 2.9	84.8 ± 4.1	76.0 ± 0.9	86.6 ± 0.3
Olin	76.4 ± 3.0	75.0 ± 3.9	78.6 ± 1.4	83.3 ± 0.0
Pitt	73.2 ± 1.8	75.0 ± 2.6	71.4 ± 6.4	72.4 ± 5.4
Leuven	74.6 ± 3.2	68.8 ± 5.4	80.6 ± 0.7	78.6 ± 0.7
UCLA	79.5 ± 3.1	77.8 ± 3.7	75.0 ± 2.3	79.2 ± 2.3
Caltech	67.6 ± 2.7	71.4 ± 0.0	62.5 ± 6.3	71.4 ± 3.6
CMU	66.7 ± 3.6	64.3 ± 7.1	69.2 ± 0.0	69.2 ± 2.2
MaxMun	65.4 ± 3.8	69.2 ± 0.3	61.5 ± 6.7	64.3 ± 4.9
SBL	66.7 ± 3.4	71.4 ± 1.2	62.7 ± 6.2	62.5 ± 4.9
SDSU	61.6 ± 2.7	60.1 ± 5.7	67.9 ± 3.8	65.6 ± 3.2
Stanford	67.7 ± 4.2	51.6 ± 5.7	76.9 ± 5.3	64.2 ± 3.7
UM	63.8 ± 5.6	70.5 ± 4.3	53.2 ± 5.7	75.6 ± 4.9
Trinity	68.8 ± 5.5	72.9 ± 4.6	60.5 ± 5.7	73.3 ± 3.7
AVERAGEE	70.6 ± 4.0	71.7 ± 4.1	69.4 ± 4.8	71.7 ± 4.0

## Discussion

### Analysis of the hypergraph learning

To evaluate that BLS plays an important role in our proposed method, we use BLS alone for the final classification task on four representative single-center data. Meanwhile, to demonstrate the improved classification accuracy of high-order brain functional connectivity networks based on hypergraph and BLS, we use BLS to classify the features obtained based on the hypergraph. In particular, we select four representative single-center datasets for comparison based on previous work (Eslami and Saeed, 2019). Our approach is compared with the following methods, as shown in Table 4.

**TABLE 4:** Classification performance of ASD identification achieved by six different methods on four datasets (*i.e.*, OHSU, NYU, USM and UCLA) with rs-fMRI data.

Site	Method	Accuracy (%)	Sensitivity (%)	Specificity (%)	Precision (%)
**OHSU**	SVM	53.8 ± 5.2	55.1 ± 6.1	48.9 ± 7.2	52.6 ± 6.7
	SVM-ATM	70.9 ± 3.7	69.9 ± 5.6	66.8 ± 4.1	70.1 ± 5.5
	MLP	64.0 ± 4.5	56.5 ± 3.9	61.6 ± 4.2	60.3 ± 4.7
	Autoencoder	74.0 ± 3.5	66.6 ± 2.9	75.5 ± 4.7	71.5 ± 4.1
	BLS	75.5 ± 5.1	66.3 ± 3.8	72.6 ± 4.9	75.3 ± 3.9
	Ours	80.8 ± 3.8	76.9 ± 7.7	84.6 ± 7.7	83.3 ± 8.3
**NYU**	SVM	57.1 ± 2.5	50.3 ± 3.5	62.2 ± 2.7	57.8 ± 3.9
	SVM-ATM	71.2 ± 5.1	53.3 ± 4.2	81.0 ± 1.2	69.1 ± 6.5
	MLP	64.3 ± 4.2	68.4 ± 3.7	60.6 ± 3.9	57.1 ± 4.3
	Autoencoder	65.7 ± 3.2	68.8 ± 2.6	63.2 ± 2.7	61.1 ± 4.8
	BLS	69.7 ± 3.5	67.4 ± 6.3	71.1 ± 1.1	70.8 ± 1.4
	Ours	71.4 ± 5.7	75.0 ± 1.5	68.4 ± 9.4	66.7 ± 9.8
**USM**	SVM	64.7 ± 5.1	60.6 ± 1.9	66.9 ± 5.1	60.7 ± 3.9
	SVM-ATM	69.6 ± 4.6	44.3 ± 3.8	68.2 ± 6.3	61.8 ± 4.3
	MLP	64.1 ± 4.1	61.2 ± 3.8	65.4 ± 4.2	62.9 ± 3.8
	Autoencoder	62.5 ± 2.8	60.0 ± 3.2	66.3 ± 4.5	62.5 ± 4.1
	BLS	76.9 ± 3.1	78.5 ± 2.9	79.8 ± 3.9	82.2 ± 3.9
	Ours	81.6 ± 2.9	84.8 ± 4.1	76.0 ± 0.9	86.6 ± 0.3
**UCLA**	SVM	65.1 ± 5.7	68.3 ± 3.5	60.8 ± 4.7	65.2 ± 3.3
	SVM-ATM	72.2 ± 3.1	73.8 ± 4.1	68.9 ± 3.8	69.2 ± 2.8
	MLP	71.9 ± 3.5	72.7 ± 2.4	64.8 ± 3.1	66.1 ± 3.2
	Autoencoder	57.7 ± 4.6	68.2 ± 4.1	47.4 ± 4.8	58.5 ± 3.9
	BLS	73.2 ± 2.8	76.4 ± 4.5	65.8 ± 4.9	71.6 ± 3.1
	Ours	79.5 ± 3.1	77.8 ± 3.7	75.0 ± 2.3	79.2 ± 2.3

(1) We first compare with the SVM method as well as the MLP method. We also compare with SVM methods that incorporated parameter tuning (i.e., SVM as a classifier using the hyperparameter tuning method Auto Tune Models (ATM)) (Eslami and Saeed, 2019).

(2) We then compare with the ASD classification method based on autoencoder and multilayer perceptron proposed by Heinsfeld et al. (2017).

To demonstrate separately that hypergraphs and BLS play an important role in our proposed method, we first used BLS alone to learn the low-order brain functional connectivity network for ASD classification, followed by the construction of a high-order brain functional connectivity network based on hypergraphs, which is then learned and classified by autoencoder and BLS. Table 4 shows the experimental results of each method, and the results show that our proposed method using only BLS to learn the lower-order brain functional connectivity network significantly outperforms traditional machine learning methods as well as recent deep learning-based methods such as autoencoder-based methods.

The model performance is further improved when the high-order brain functional connectivity network is represented using hypergraphs. Therefore, our proposed method demonstrates significant superiority over other methods.

### Analysis of the broad learning system

**FIGURE 3:**
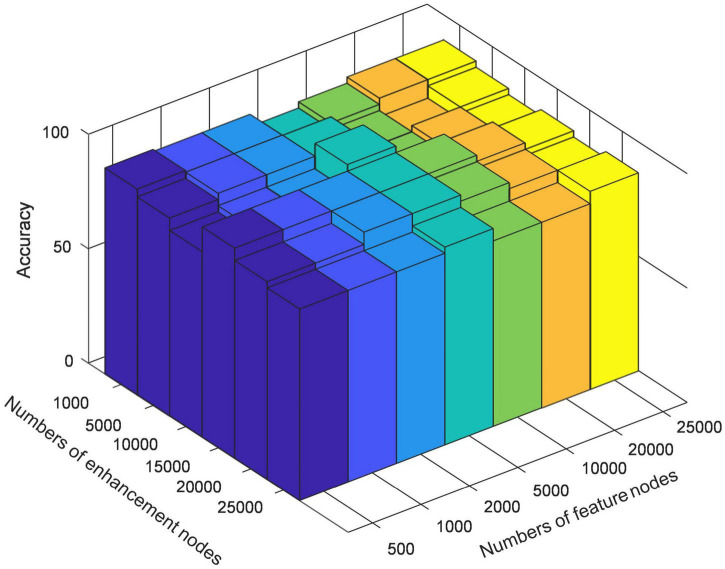
The classification accuracy on different mapping features settings.

In order to verify the effectiveness of BLS in further extracting discriminative features of high-order brain functional connectivity networks and optimize the specific structure of BLS, we test the classification results of different mapping features of multi-center data. We empirically set the initial number of enhancement nodes to 1,000, 5,000, 10,000, 15,000, 20,000, 25,000, respectively, and then gradually increase the number of mapping nodes in steps of 500. Figure 3 shows the variation in model performance at some typical nodes settings during optimization. BLS has obtained classification results with high accuracy under the settings of different mapping nodes. In most cases, when the number of enhancement nodes is fixed, model performance becomes better and worse when the number of mapping nodes continuously increases. Therefore, the optimal node setting can be found in this process. When the number of mapping nodes and the number of enhancement nodes reaches 5,000 and 10,000, respectively, the best result, 76.2%, can be obtained.

### Analysis of high-order brain connectivity network

**FIGURE 4:**
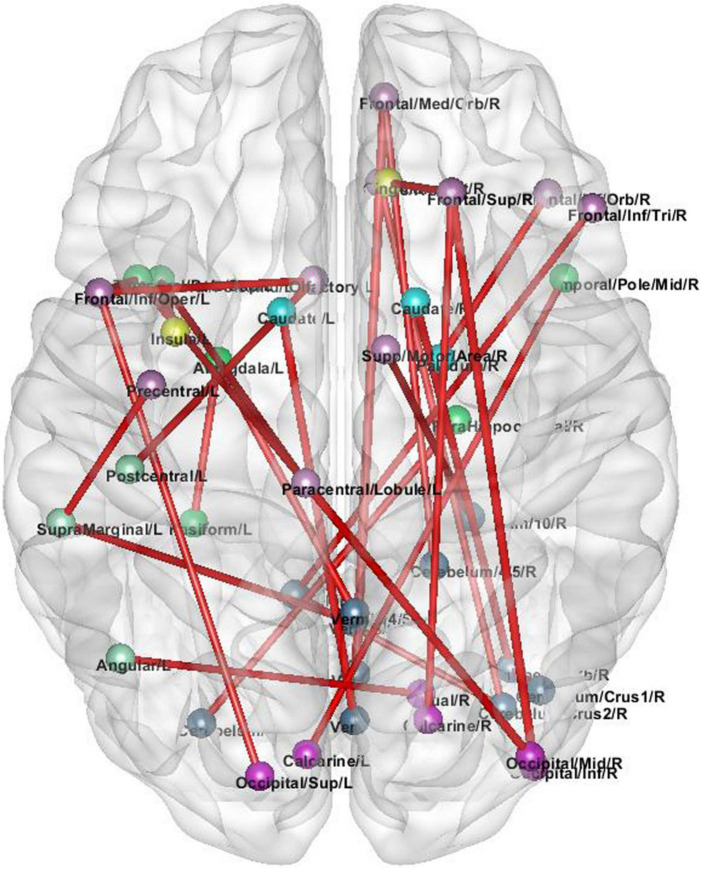
The selected high-order brain functional connections.

In order to explore the high-order connections associated with ASD in the brain functional connectivity network, we analyze the high-order brain functional connections that are selected most frequently. As shown in Figure 4. These ROIs are colored according to the static network to which they belong. We find the selected brain connections and regional distribution of brain regions scatter across the two hemispheres and different lobes, showing a pattern of functional abnormalities throughout the brain of ASD patients. Specifically, the top brain FCs are visualized in Figure 4. The connections in Red represent the edges of brain ROIs and the ROIs with same color belong to the same brain modules. The brain regions shown in Figure 4 are highly associated with ASD (Wang et al., 2014). The selected connectivities include the salience network and cerebellum region, and these regions are also shown to be closely related to ASD. These results verify the reliability of our proposed method in detecting informative functional connectivity for ASD identification.

## Experiments on other brain atlas

To verify the robustness and scalability of our method on another atlas, we further select the ABIDE dataset for our experiments, using the preprocessing method and brain region segmentation method used by Mostafa et al. (2020) and Yin et al. (2022). In contrast to the aforementioned segmentation of brain regions into 200 ROIs based on the cc200 atlas, we segment the brain into 264 ROIs and then obtain another brain feature representation by calculating the high-order brain functional connectivity network among the 264 ROIs, *i.e.*, we obtain 69,432 pairwise correlation features. We use 871 samples from the ABIDE dataset as in Mostafa et al. (2020); Yin et al. (2022) for our experiments. We compare our proposed method with the latest methods, namely (1) the autoencoder based method for ASD diagnosis proposed by Eslami and Saeed (2019); Eslami et al. (2019), (2) a novel convolutional neural network method proposed by Xing et al. (2019), (3) an autoencoder and DNN classifier based method for ASD diagnosis proposed by Mostafa et al. (2020), and a method based on logarithmic Euclidean and affine invariant Riemann metric connectivity matrices proposed by Wong et al. (2018). Table 5 shows the algorithm we compared with and the experimental results. Experiments confirm that our proposed method is significantly superior to other methods in characterizing functional connectivity relationships in the brain. Compared to the autoencoder-based methods proposed by Mostafa et al. (2020) and the Euclidean and affine-invariant Riemannian metric-based connectivity matrix-based methods proposed by Wong et al. (2018), our method performs well on another brain atlas. We experimentally demonstrate the robustness and scalability of our method.

**TABLE 5:** Comparison with the state-of-the-art methods using other brain atlas.

Method	Accuracy (%)	Sensitivity (%)	Specificity (%)	Precision (%)
ASD-DiagNet (Eslami et al., 2019)	68.4 ± 1.2	70.9 ± 0.5	65.7 ± 2.6	65.2 ± 3.5
CNN + Element-wise Filters (Xing et al., 2019)	65.7 ± 3.8	70.8 ± 0.1	61.3 ± 7.0	61.3 ± 7.4
Auto-AsD-Network (Eslami and Saeed, 2019)	70.1 ± 1.7	71.7 ± 0.3	68.5 ± 2.8	70.5 ± 5.3
Autoencoder + DNN (Mostafa et al., 2020)	79.1 ± 1.8	77.5 ± 5.8	80.7 ± 12.5	80.0 ± 10.5
Riemannian Regression (Wong et al., 2018)	71.1 ± 1.5	72.7 ± 0.8	69.4 ± 2.9	71.1 ± 4.8
Ours	83.1 ± 3.9	82.2 ± 5.1	80.7 ± 4.2	86.0 ± 6.3

## Conclusion

We propose a deep-broad learning-based method to explore the high-order brain functional connectivity for ASD diagnosis. Our hypergraph-based higher-order brain functional connectivity network helps to characterize the global features of the brain. The use of autoencoder and BLS to sequentially learn high-order features makes the ASD detection model more efficient and effective. Our experiments are conducted on single-center and multi-center data of the ABIDE dataset. To verify the robustness and scalability of the method, we perform additional experiments on another brain atlas that divide brain regions into 264 ROIs. Experimental results demonstrate that our profiled high-order brain functional connectivity network can represent more discriminative global brain features. The combination of BLS and autoencoder further quickly learns the features, and the diagnostic model can achieve higher accuracy.

## Data availability statement

The datasets analyzed for this study can be found in the ABIDE-I repository in Craddock et al. (2013) (http://preprocessed-connectomes-project.org/abide/).

## Author contributions

XH: supervision and editing. QA: methodology, writing—original draft, and coding. JL: methodology and coding. HM: writing—original draft. YG: investigation. MY: supervision. JQ: supervision. All authors contributed to the article and approved the submitted version.
